# Mimicking Molecular Pathways in the Design of Smart Hydrogels for the Design of Vascularized Engineered Tissues

**DOI:** 10.3390/ijms241512314

**Published:** 2023-08-01

**Authors:** Aldo Nicosia, Monica Salamone, Salvatore Costa, Maria Antonietta Ragusa, Giulio Ghersi

**Affiliations:** 1 Institute for Biomedical Research and Innovation-National Research Council (IRIB-CNR), Via Ugo la Malfa 153, 90146 Palermo, Italy; monica.salamone@cnr.it; 2 Department of Biological, Chemical and Pharmaceutical Sciences and Technologies (STEBICEF), University of Palermo, Viale delle Scienze, Ed. 16, 90128 Palermo, Italy; salvatore.costa@unipa.it (S.C.); maria.ragusa@unipa.it (M.A.R.); giulio.ghersi@unipa.it (G.G.)

**Keywords:** angiogenesis, biomaterials, hydrogel, molecular signaling, regenerative medicine, gene signature

## Abstract

Biomaterials are pivotal in supporting and guiding vascularization for therapeutic applications. To design effective, bioactive biomaterials, understanding the cellular and molecular processes involved in angiogenesis and vasculogenesis is crucial. Biomaterial platforms can replicate the interactions between cells, the ECM, and the signaling molecules that trigger blood vessel formation. Hydrogels, with their soft and hydrated properties resembling natural tissues, are widely utilized; particularly synthetic hydrogels, known for their bio-inertness and precise control over cell–material interactions, are utilized. Naturally derived and synthetic hydrogel bases are tailored with specific mechanical properties, controlled for biodegradation, and enhanced for cell adhesion, appropriate biochemical signaling, and architectural features that facilitate the assembly and tubulogenesis of vascular cells. This comprehensive review showcases the latest advancements in hydrogel materials and innovative design modifications aimed at effectively guiding and supporting vascularization processes. Furthermore, by leveraging this knowledge, researchers can advance biomaterial design, which will enable precise support and guidance of vascularization processes and ultimately enhance tissue functionality and therapeutic outcomes.

## 1. Introduction

Regeneration of tissues after injury states as well as after transplantations and implanting mainly relies on rapid vascularization, which ensures proper oxygen and nutrient transport to cells. To obtain such a result, the implementation of vascularization units in tissue engineering approaches for regenerative medicine represents an interesting option. In addition to therapeutic implantation, various fundamental and applied investigations demonstrate the application of artificially created tissues to develop pharmacological tests in a laboratory setting before their clinical implementation. This approach is particularly relevant in the context of personalized therapies, as well as in the utilization of model tissues, such as organs on a chip [1]. These engineered tissues serve as valuable tools for examining alternative regeneration techniques and exploring the interplay between various cell types [2].

In addition, several studies addressed the possibility to repair tissue defects and injuries via tissue engineering applications [3,4,5,6]. For engineered tissue systems that exceed the limit of diffusion (~200 µm [7,8]), the implementation of a functional network of blood vessels represents a required step since it is mandatory that some vascularization strategies need to be used to ensure oxygen and nutrient supplies to all the cells of the system.

Because natural or synthetic hydrogels possess mechanical features resembling soft tissues, they are usually exploited as supporting scaffolds for tissue engineering applications; they also provide modifications intended for promoting the formation of stable and permeable vascular networks throughout the engineered tissues [9,10].

It is generally known that two distinct processes, namely vasculogenesis and angiogenesis, are responsible for the generation of blood vessels. A finely regulated sequence of stimuli (including diffusible molecules, cell–cell contacts, and interactions) is required to achieve the formation of a permeable network. While the creation of an initial vascular locus starting from recruited cells during embryogenesis is known as vasculogenesis, the sprouting of new vessels from pre-existing blood vessels is defined as angiogenesis [11,12]. Vascularization of tissue may also be achieved via other mechanisms including intussusception and vessel co-option, which give rise to daughter vessels from pre-existing vessels; however, these mechanisms are far from being well-defined, even though they are commonly used to vascularize tumor tissues [13].

Topic cues in the application of regenerative strategies come from the vascular niche surrounding the blood vessels which include the composition of the ECM and the interplay among vascular and non-vascular cells, as well the contribution from stem and mesenchymal cells [14]. Thus, efforts were made to integrate cues coming from niche components within biomaterial platforms to promote strategies of therapeutic vascularization in regenerative medicine protocol such as islet transplantation [15,16,17], neuronal [18], and musculoskeletal regeneration [19,20]. Interestingly, engineered vascularizing biomaterials have also been used for the development of perfused angiogenic models for the study of cancer progression and treatments [21,22,23].

In order to recapitulate the angiogenesis in vitro, the used scaffold needs to encompass the main physiological aspects because all the involved cells, including smooth muscle cells’ pericytes and endothelial cells, have to interact with the perivascular synthetic niche and degrade it in a time-dependent manner; the degradation must occur according to the finely regulated expression of metalloproteases (MMPs) required for the initial degradation of the basal lamina and for the remodeling of the extracellular matrix (ECM) [24,25,26,27].

Hence, the selected biomaterials should be designed taking into account cell adhesion, migration, and biodegradation properties of the scaffold, thus tuning cell adhesiveness, mechanical properties, and stability of the used platforms. Moreover, it is recognized that the “switch on” towards an angiogenic fate primarily relies on the overexpression of pro-angiogenic factors and/or downregulation of angiogenic inhibitors. Indeed, several growth factors (GFs) including VEGFs, FGF, and PDGF activate diverse signaling pathways that, in a scheduled manner, regulate several aspects of angiogenesis [28,29]. Thus, it is not unexpected that vascularization for tissue-engineered applications is also achieved using smart biomaterials; these materials present angiogenic factors incorporated into scaffolds or ensure the sequential/simultaneous delivery of different GFs [30,31,32]; in turn, this may serve to model the vessel architecture in the engineered constructs.

In this review, we recapitulate the main process regulating angiogenesis, while considering the GFs, ECM, and signaling pathways in the perivascular niche that are eligible to be integrated into biomaterials to properly support vascularization. Moreover, we delineate the general strategies for the development of vascularized engineered tissues either using naturally derived biomaterials such as collagen, gelatin, hyaluronic acid (HA) fibrin, and decellularized ECM for a top-down approach; and synthetic materials such as poly(vinyl alcohol) (PVA) and poly(ethylene glycol) (PEG), which are usually applied in bottom-up strategies following functionalization. Finally, we discuss the need for combining molecular biology, biochemistry, cell biology, and microfluidic expertise in the integration of angiogenic niche physiology within smart hydrogels.

## 2. Molecular Signaling Pathways Involved in the Angiogenic Process

**Figure 1:**
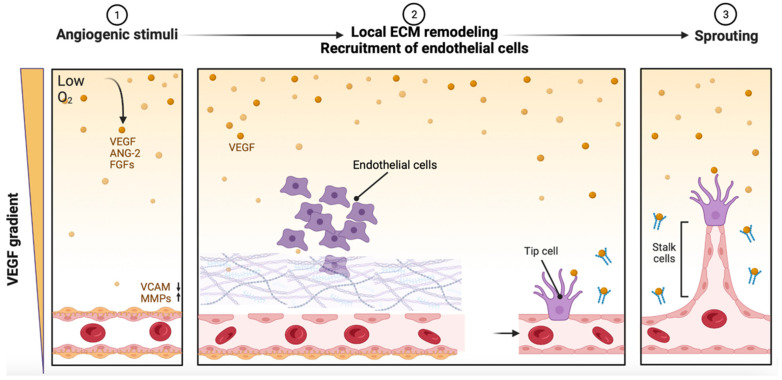
Angiogenesis occurs in response to different conditions such as lowering the O_2_, which induces VEGF increase in tissues. VEGFs bind and promote tip cell differentiation, loosening cell contacts and increasing the expression of MMPs, while simultaneously inhibiting tip cell formation in adjacent cells via notch signaling. Remodeling of the ECM mediated by MMPs and recruitment of ECs are fundamental for proper sprouting.

As written above, two main processes account for the vascularization of tissues: vasculogenesis, mainly occurring during embryonic development, and angiogenesis, which relies on the remodeling of vasculature via the sprouting of new vessels from pre-existing vessels [11,13,29]. Although these processes also show overlapping mechanisms, because vasculogenesis is usually uncommon in adult tissues, we herein limit the discussion of angiogenesis. During the angiogenic process, schematically reported in Figure 1, the remodeling of existing vasculature occurs in several steps: breakdown of the vessel wall and remodeling of the surrounding ECM; recruitment of activated endothelial cells (ECs), which proliferate and invade the surrounding tissue; recruitment of pericytes and mesenchymal stem cells, stopping the ECs’ proliferation and organizing the vessel; deposition of the basement membrane; and finally, formation of a new perfusable vessel [33,34,35,36,37,38].

In healthy adult vessels, quiescent ECs form a monolayer of cells known as phalanx cells that are surrounded by pericytes; these cells suppress EC activation/proliferation and stabilize the vessel. Angiogenesis is established in response to VEGF-Cs, ANG-2s, and FGFs released after injury, inflammation, or hypoxic signals [39,40,41,42,43,44,45]. A downregulation of vascular cell adhesion protein (VCAM-1) caused by a VEGF leads to the loosening of vessel cell junction, while pericytes, in response to ANG-2, induce the proteolytic ECM degradation of the basement and are mediated by MMPs [28,46]. It is interesting to note that VEGFs also act to induce changes in the permeability of the vessel, allowing plasma proteins to extravasate and depose a temporary provisional extracellular matrix (ECM) scaffold. Due to the interaction of integrins and the ECM, recruited ECs migrate onto this ECM surface.

Protease activity leads to the release of angiogenic molecules like VEGFs and FGFs from the extracellular matrix (ECM) and reshapes the ECM to create a conducive environment for angiogenesis. During angiogenesis, a single endothelial cell, known as the tip cell, is selected to lead the formation of a perfused tube, while other neighboring endothelial cells assume subsidiary roles as stalk cells [13]. The tip cell is guided by factors such as VEGF receptors, neuropilins (NRPs), and NOTCH ligands (DLL4 and JAGGED1) [28,47], while the stalk cells elongate the stalk, establish the lumen, and convey positional information through various signaling mechanisms (including NOTCH, NRARP, WNTs, PDGF, FGFs, VE-cadherin, CD34, sialomucins, VEGF, and hedgehog) [48]. Tip cells possess filopodia to sense guidance cues from the environment, while stalk cells release molecules like EGFL7 into the ECM to support stalk elongation [49]. Hypoxia-inducible programming, regulated by HIF-1α, enables endothelial cells to respond to angiogenic signals. Myeloid bridge cells aid in vessel fusion, facilitating blood flow initiation.

To achieve functionality, vessels must mature and stabilize. Endothelial cells transition to a quiescent state, aided by signals such as platelet-derived growth factor B (PDGF-B), ANG-1, TGF-β, ephrin-B2, and NOTCH, resulting in pericyte coverage [8,13]. Protease inhibitors, such as tissue inhibitors of metalloproteinases (TIMPs) [25,27] and plasminogen activator inhibitor-1 (PAI-1), promote the deposition of a basement membrane, while junctions are reestablished to ensure optimal blood flow distribution [50,51,52]. Vessels undergo regression if they fail to become perfused.

Throughout the angiogenesis process, nearby and enlisted endothelial cells create fresh tissue barriers, leading to vessel splitting or the arrangement of specific cell types known as stalk and tip cells that are responsible for the sprouting. Growing vessels stretch their edges towards VEGF gradients. Nonetheless, to hinder unregulated angiogenesis, endothelial cells themselves instigate the subsequent phase by discharging platelet-derived growth factor (PDGF) [11,41,53]. Mural cells are recruited by PDGF, which induces their differentiation into pericytes and smooth muscle cells. They will surround the endothelial tubules, leading to the formation of mature vessels [36,39]. Through endothelial cell–mural cell contact, the vascular networks activate transforming growth factor-β (TGF-β), which facilitates ECM reestablishment, growth arrest, and terminal differentiation of vascular cells. This regulatory mechanism controls the extent of vascularization and prevents excessive and uncontrolled angiogenesis.

The complex process of vascularization involves the regulation of various signaling factors, including Angiopoietin-1 and -2 (Ang-1 and Ang-2) and endoglin. The molecular signaling pathways commonly involved in vascular development are summarized in Table 1. Ang-1 is primarily associated with vessel maturation, while Ang-2 acts as a vessel remodeler by promoting the detachment and loosening of vascular cells during angiogenesis initiation [34,35,45,46]. TGF-β, known for its involvement in vessel maturation, exhibits diverse effects on vascular cell behavior depending on the context, necessitating careful control of its presentation. Endoglin, a coreceptor, is expressed by ECs; endoglins regulate the activity of TGF-β [54] by influencing its binding to ALK-1 and ALK-5 [36,55].

Environmental cues play a significant role in influencing the expression, stability, and activity of vascular growth factors. In tissues experiencing hypoxia, where there is insufficient oxygen, the release and stimulation of pro-angiogenic factors are triggered [35]. Hypoxia-inducible factor-1 acts as a specific pivot, either in the upregulation or in the stabilization of VEGF mRNA [29,39,41]. Conversely, hyperoxia inhibits the expression of VEGFs, leading to the regression and demise of blood vessels [29]. As endothelial tubules mature, blood perfusion prompts endothelial cells to release PDGFs. This, in turn, targets mural cells expressing the PDGF receptor, facilitating adhesion to the endothelial tubes [29,56,57,58]. Vascularization is a dynamic and complex process involving multiple components. A comprehensive understanding of the biological mechanisms governing vessel formation is crucial for incorporating relevant cues into biomaterial systems. This understanding enables the optimization of vascularization outcomes in such systems.

**Table 1:** Molecular signaling pathways commonly involved in vascular development.

Pathway	Activity	References
VEGFs	Regulation on endothelium proliferation, mural permeability, crosstalk, EC recruitment.	[41,42,43]
ANG1/2/4	Regulation of endothelium permeability and sprouting regulation.	[44,45]
NOTCH1/4	Sprouting regulation	[28,47]
VCAM	Regulation of endothelium adhesion and downregulation induced by VEGFs causes vascular permeabilization	[59,60]
WNT	Regulation of NOTCH signaling pathway.	[61]
PDGF	Recruitment of mural cells and promotes their differentiation into pericytes.	[53]

## 3. Integrating Signaling Pathways in the Design of Smart Hydrogels

**Figure 2:**
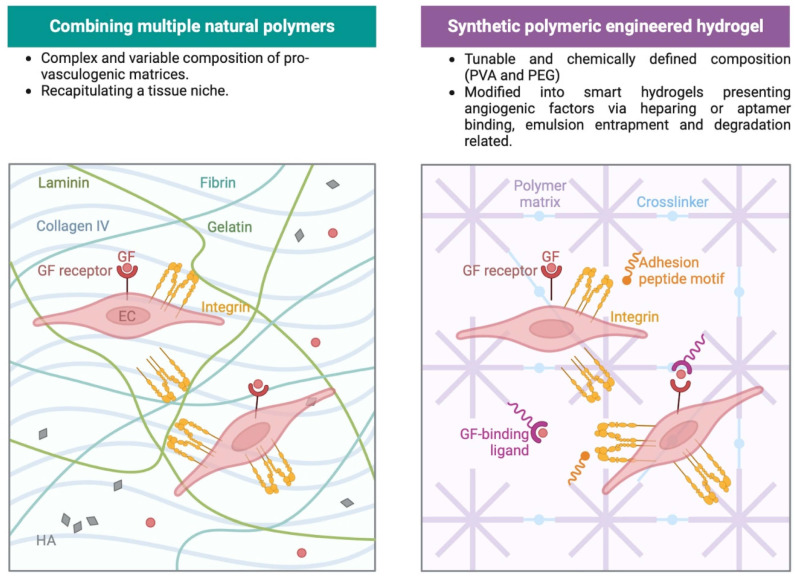
The hydrogel composition plays a pivotal role in angiogenic development and in the ability to remodel a vasculature network. Both naturally derived and synthetic polymers are commonly used to support in vitro and in vivo angiogenesis. Presentation of angiogenic factors is usually achieved via soluble supplementation, transient sequestration, and covalent binding to hydrogel components. More details are reported in the text.

Hydrogel biomaterials have been employed to facilitate the process of vascularization. These materials are designed to provide a supportive environment for the growth and development of blood vessels. By incorporating specific cues and factors, hydrogel biomaterials can effectively promote angiogenesis, allowing for the formation of a functional vascular network. The properties of hydrogels, such as their biocompatibility and tunable physical characteristics, make them suitable for creating a conducive microenvironment that supports vascular cell proliferation, migration, and organization. Through careful design and optimization, hydrogel biomaterials hold promise in advancing strategies for successful vascularization in various biomedical applications. Numerous hydrogel culture platforms are available, offering a variety of options for tissue growth. These platforms can be fabricated using natural or synthetic polymers, or a combination of both (Figure 2). In addition, for injectable hydrogel, it is also possible to achieve gelation in a stimuli-responsive fashion which is thought to be related to the surrounding environment [62].

### 3.1. Naturally Derived Hydrogels

Hydrogels made from natural polymers, such as collagen, gelatin, fibrin, and hyaluronic acid (HA), are commonly used in this context [1,63,64,65,66,67]. Natural polymer hydrogels possess innate and proper cell interaction activities achieved through receptor–ligand binding and can be biodegraded enzymatically, making them advantageous for supporting vascularization [63,65]. Consequently, they have been extensively employed in studying vascularization processes and facilitating the growth of blood vessels. Naturally derived hydrogels form through self-assembly physical crosslinking, a process which involves changes in intermolecular interactions. Gelation is achieved by modifying the temperature (increasing to 37 °C or decreasing to −20/−80 °C). Several parameters, including temperature, pH, and ionic strength, can be controlled to achieve the desired hydrogel structure, while chemical and physical crosslinking combinations are often applied. Table 2 reports principal gelation methods that are usually used.

Among natural polymers, hyaluronic acid (HA) is a versatile biomaterial widely used in tissue engineering and regenerative medicine. With its biocompatibility and ability to retain water, it forms a three-dimensional scaffold that mimics the native extracellular matrix [65]. Hyaluronic acid hydrogels promote cell adhesion, migration, and proliferation, making it suitable for applications in wound healing, drug delivery, and cartilage regeneration. Its tunable properties and bioactive modifications contribute to its therapeutic potential. HA is a prominent component of the natural ECM. It possesses high hydrophilicity and biodegradability [68,69,70,71,72]. The biological exploitation of HA mainly relies on the molecular weight: HA with a high molecular weight (approximately 10^6^ Da) is nonimmunogenic and exhibits antiangiogenic properties, while low molecular weight HA (less than 3.5 × 10^4^ Da) exerts pro-angiogenic activity but can also induce inflammation by activating APC also via chemokines [73,74]. Consequently, HA hydrogels designed to facilitate controlled vascularization are typically composed of high molecular weight HA and modified to enhance angiogenesis.

Collagen, which is the most abundant protein found in the ECM [75], is widely utilized as a natural polymer in biomaterials. Collagen gels offer cell adhesion, cell spreading, and enzymatic degradation properties, thus meeting the fundamental requirements for vascularization support, other than the necessary control of stiffness [76,77,78,79,79,80,81]. A hydrogel known as HA-KLT was developed by modifying hyaluronic acid (HA) with a VEGF mimetic peptide called KLT (KLTWQELYQLKYKGI). Characterization of the hydrogel revealed a porous, three-dimensional scaffold structure that offered a large specific surface area for cell adhesion and interaction. In comparison to the unmodified HA hydrogel, the HA-KLT hydrogel demonstrated enhanced capability in promoting the attachment, spreading, and proliferation of endothelial cells in vitro. Additionally, the pro-angiogenic potential of the hydrogels was assessed by implanting them into lesion cavities in injured rat brains. Results showed that the hydrogels were able to establish a permissive interface with the host tissues after four weeks of implantation [82].

Gelatin, derived from collagen through acid or base treatment, is another commonly used natural polymer in biomaterials due to its affordability, degradability by cell-secreted proteases, and stability under various conditions [65,83,84,85]. In addition, the physical and mechanical properties of gelatin hydrogels can be finely tuned via the crosslinking of type-A and type-B gelatin catalyzed by microbial transglutaminase via reactive methacryloyl groups; thus, gelatin can be transformed into gelatin methacrylate (GelMA) and subsequently crosslinked to form hydrogels that promote vascularization [86,87,88,89,90,91]. In addition, GelMA mechanical properties may be tuned by varying the degree of methacrylamide groups [92,93].

Fibrin, a protein formed from the breakdown of fibrinogen by thrombin during coagulation, serves not only as a hemostatic agent but also as a temporary matrix during the initial stages of wound healing [94,95,96]. It supports the invasion and adhesion of endothelial cells, facilitates the vascularization of wound sites, and acts as a reservoir for pro-angiogenic growth factors [97]. It was successfully used to promote anastomosis in vitro by the coculturing of endothelial cells (ECs) and fibroblasts in a fibrin 3D gel [98]. In vitro, a hydrogel material can be created by mixing fibrinogen with thrombin and calcium ions [99,100,101]. Fibrin-based hydrogels, renowned for their ability to promote vasculogenesis, are commonly employed in various models of vascularization due to their ease of fabrication [99,100,102,103,104,105]. Furthermore, fibrin can be recovered from blood for the creation of autologous hydrogel, which ensures that viable implants can be used therapeutic applications [106].

When aiming to create ECM-protein-based matrices for vascular tissue generation, it is beneficial to mimic the basement membrane of the native vascular environment. The basement membrane consists mainly of laminin, collagen, perlecan, nidogen, and smaller amounts of fibronectin [107,108,109]. Therefore, when designing pro-vasculogenic matrices, it is common to incorporate laminin along with other natural polymers, leading to improved angiogenic properties [110,111]. For instance, it has been shown that the incorporation of laminin within a collagen hydrogel enhances vessel formation when cells may make contact with it. This combination promotes cell adhesion, increases VEGFR expression, and facilitates the formation of endothelial networks [112]. Therefore, as a general rule, the combination of different natural polymers will provide a variety of signals which enhance vascularization [112,113,114,115,116,117]. Blending different natural polymers allows for optimal combinations of their advantageous properties (Figure 2). For example, combining collagen I with GelMA improves not only the mechanical properties of the hydrogel but also ensures improved vascularization due to the activation of additional molecular signaling pathways [114,115]. Other used combinations showed that the inclusion of fibrin, which contributes angiogenic signaling, within HA hydrogels improves scaffold longevity and supports vessel formation; mixing with chitosan also provides the same effects [118,119,120]. This has been reported to induce biodegradability, provide easy modification procedures, improve mechanical properties, and, in combination with gelatin, enhance ECM-related signaling [121,122].

The most accurate representation of the natural cellular environment is achieved with decellularized ECMs, a natural hydrogel material [123]. After decellularization, proteins and polysaccharides of the ECM still remain, offering a tissue-mimetic architecture experienced in vivo to the englobed cells [108]. Decellularized ECMs have been found to promote greater angiogenesis compared to collagen alone, as it also provides other ECM-related signaling [124,125]. It has been shown that the use of decellularized scaffolds mainly consisting of collagen and elastin, when seeded with ECs, are able support angiogenesis both in vitro and in vivo [125,126]. Similarly, retaining collagen and laminin in ECM-based scaffolds together with adipose-derived stem cells or microvascular fragments will enhance therapeutic approaches for the treatment of acute myocardial infarction [1,127,128,129].

As an advantage, this approach provides natural biocompatibility, the absence of toxicities, and the activation of several signaling pathways. However, several problems due to the simultaneous activation of different signals occur when aiming to study specific cell–ECM constituent interactions. Additionally, variations in the procedure of isolation from different provenances hinder the reproducibility because of large batch-to-batch variability [64].

**Table 2:** Naturally derived hydrogel preparation.

Gelation Method	Biomaterials	References
Crosslinkers	HA	[130]
Temperature increase	Collagen	[131]
Crosslinkers	Gelatin	[132,133]
Cation adding	Fibrin	[134]
Temperature decrease		

### 3.2. Synthetic Polymer Hydrogels and Modifications to Promote the Angiogenesis

While some naturally occurring polymer frameworks have shown potential in promoting vascularization, they do not provide a thoroughly regulated and precisely defined setting to investigate the impact of environmental signals on cell behavior. On the other hand, synthetic polymer hydrogels offer a highly customizable material, and they have been applied in different settings other than angiogenic ones [135,136,137]. However, they require significant modifications to mimic the natural environment of cells and interact effectively with them. Unlike naturally derived ones, the use of synthetic hydrogels may also account for the resulting cytotoxicity mainly due to the procedure of gelation or crosslinking. Common synthesis methods used for polymer hydrogel production are reported in Table 3. Biocompatibility is crucial for polymer gel components and gelation processes in tissue engineering applications. For instance, poly(acrylamide)-based gels have been employed in the past due to their tunable mechanical stiffness of the support [138,139,140]. Nonetheless, their utilization is restricted to 2D cell culture due to the toxic nature of acrylamide monomers before polymerization. Therefore, if cells are to be encapsulated in a 3D environment, biocompatible synthetic gels must be employed.

Apart from diminishing toxicity, synthetic gels should also aim to decrease inflammatory responses within a living organism. This objective can be accomplished by employing a bioinert chemical composition that discourages protein adsorption, a feature commonly found in numerous synthetic hydrogel-forming polymers like poly(2-hydroxyethyl methacrylate) (PHEMA) and poly(vinyl alcohol) (PVA) [63]. Among bioinert synthetic polymers, poly(ethylene glycol) (PEG) is the most widely used polymer in tissue engineering. PEG not only wards off protein adsorption due to its hydrophilicity and chain pliability, but also lacks hydrogen-bond-donating moieties, rendering it more impervious to protein adsorption in contrast to PHEMA and PVA [63]. While bio-inertness has been considered to be required for preventing undesired reactions including uncontrolled cell–protein interactions and foreign body responses, it also causes some limitations, such as in its impacts on interactions with cells and its influence on tissue support. Therefore, a tailored approach based on the integration of peptides and proteins is usually required to overcome such limitations.

This allows for the development of custom networks capable of executing specific functions like precise cell attachment, degradation rate control, and proper spatiotemporal presentation of angiogenic factors.

Artificial hydrogels have been extensively studied and display minimal batch-to-batch variability. They have found widespread use in promoting vascularization in both laboratory settings (in vitro) and in living organisms (in vivo). For example, gels formed from a blend of polymers based on polyvinyl alcohol (PVA) have been employed to investigate promoting vascularization [141,142,143]. However, hydrogels based on PEG are widely utilized and have consistently shown reliable support for vascularization when suitably modified [144,145,146].

**Table 3:** Common crosslinking methods and synthetic hydrogels in angiogenic applications.

Synthesis	Hydrogel	References
Free radical polymerization via UV-sensitive initiator	PHEMA	[147]
Freeze and thaw cycling	PVA	[148]
Free radical polymerization via redox/thermalphotoinitiators	PEG	[149]

Chwalek et al. [150] developed an innovative approach to stimulate angiogenesis by incorporating heparin into PEG hydrogels, effectively sequestering VEGF, bFGF, and SDF1α. The inclusion of this component enables the regulated and prolonged release of growth factors over a period, facilitating the administration of a singular dose that proves to be effective at continuously delivering soluble growth factors throughout the culture or regeneration duration. As stated above, the reduction of adverse cell behaviors and inflammatory responses is usually achieved via the minimization of uncontrolled protein adsorption to synthetic biomaterials; however, this, in turn, may hinder cell adhesion to the hydrogel.

To achieve controlled vascularization, it is desirable to have spatiotemporal control over the presentation of pro-angiogenic factors, ensuring sustained local exposure. While soluble delivery of pro-angiogenic growth factors has shown some success in driving vascularization, a higher level of control can be achieved by permanently immobilizing these factors within the hydrogel matrix [151,152,153,154]. A widely adopted technique for attaching bioactive molecules involves covalent linking, establishing a lasting bond between the bioactive molecule and the polymer chains in the hydrogel matrix. This approach has been extensively utilized to modulate cell behavior, including directing cell phenotype and promoting stem cell differentiation [153,155]. Effective strategies supporting this approach include light-triggered, free-radical-mediated linking and click chemistry reactions (Table 4). Free-radical-mediated tethering encompasses joining biomolecules to a vinyl-modified polymer which creates crosslinks with the main polymer during network formation. In click reactions, the biomolecule is attached to a functional group that binds independently to the crosslinking pattern. The covalent attachment of angiogenic factors to hydrogels has demonstrated the activation of pro-angiogenic differentiation, both in laboratory settings and in living organisms [156,157,158].

For instance, upon covalent linking of VEGFs to a PEG gel through free radical crosslinking, notable enhancements in endothelial tubule formation in 2D culture and augmented migration of endothelial cells and cell–cell connections in 3D encapsulation are exhibited [153].

Similarly, PDGF-BB, responsible for vessel maturation, was tethered within PEG hydrogels, providing evidence for increased mature vessels either in 2D or 3D systems. The addition of covalently tethered bFGFs further increased endothelial cell migration [159]. In addition, it has been shown that gels containing both soluble and tethered PDGF-BB exhibited a significant increase in endogenous vessel ingrowth when compared to gels with only soluble PDGF-BB.

Click-chemistry-based tethering of VEGFs has also shown angiogenic behavior, with VEGFs immobilized in agarose and PEG gels leading to endothelial tubule formation in either in vitro or in vivo settings [150,160].

Although proteins like VEGF, bFGF, and PDGF have shown efficacy in enhancing vascularization, peptides present unique benefits, including their compact size, stability, and customizable manufacturing process. Peptides can be tailored to present essential regions of the protein to elicit desired cell responses while minimizing immunogenic reactions and preserving bioactivity when bound to hydrogels [161]. For example, the VEGF-mimetic peptide Qk, which consists of the 17–25 helix region of the VEGF protein, can bind the corresponding receptors on vascular ECs, stimulating their proliferation and angiogenesis [151]. Covalently linking Qk to various polymer hydrogels causes proliferation and outgrowth of ECs from spheroids, increased expression of phosphorylated VEGFR2, and enhanced vessel formation [162,163]. Immobilized Qk in combination with soluble VEGF has demonstrated the most robust angiogenic response, surpassing the performance of tethered VEGF alone [153]. This combination approach mimics the natural tissue environment and promotes the greatest vessel density and branching.

**Table 4:** Common methods for covalently tethering growth factors hydrogels.

Hydrogel Type/Tethering	Angiogenic Factors	References
PEG/free radical and click chemistry mediated	VEGF	[153]
PEG/free radical mediated	PDGF	[159]
PEG/free radical mediated	PDGF + bFGF	[159]
PEG-gelatin/click chemistry mediated	Qk	[163,164]
GelMA-nanoliposomes/encapsulation	Qk	[163,164]

#### 3.2.1. Exploiting Cell Adhesion

Adhesion of vascular cells to their substrate is crucial for their survival, as well as for spreading, migration, and cell–cell contacts. Within their native tissue environments, vascular cells directly attach to ECM proteins via integrin receptors. This attachment not only impacts their spreading and migration but also provides the sequestration of angiogenic factors and enhancement of protease expression, which, in turn, remodels the ECM. As a result, the interaction between cells and the surrounding matrix holds great significance in influencing cell behavior, including proliferation, differentiation, and the formation of blood vessels. Naturally derived hydrogels, like those composed of gelatin, collagen, fibrin, GelMA–collagen mixes, and collagen–laminin mixes, possess inherent cell-adhesive ligands that interact with receptors on endothelial and mural cells. These hydrogels promote cell adhesion, spreading, and even support the development of tubular structures when in contact with vascular cells.

Incorporating ECM constituents into synthetic hydrogels is usually associated to improved cell adhesion; however, the use of full length proteins may result in an undefined presentation of adhesive domains. To address this, a strategy is represented by the use of short peptides that can be bound to the matrix. These are designed to solely possess specific binding domains, offering greater control and stability across a broader range of conditions. These cell-adhesive peptides provide precise control over the presentation and density of adhesive sequences without introducing additional signals to the gel matrix.

Commonly used adhesive peptides, reported in Table 5, include fibronectin-derived RGD, which binds to integrins such as α5β3 and α5β1 found in various cell types including vascular cells [165,166]. Other peptides like REDV and KQAGDV, derived from fibronectin, facilitate the adhesion of vascular endothelial cells and vascular smooth muscle cells, respectively [167]. Laminin-derived peptides such as IKVAV and YIGSR bind to specific laminin receptors [168,169]. RGD, in particular, is widely utilized in hydrogel design for tissue engineering due to its presence in multiple ECM proteins and its ability to bind to integrin receptors expressed by various cell types, including fibroblasts, neural cells, and vascular cells [170,171]. Studies have shown that RGD-mediated vascular cell spreading promotes endothelialization, tubulogenesis, and vascular sprouting [172,173,174]. Similarly, IKVAV has demonstrated its supportive role in angiogenesis and wound healing [175].

The synergy between the hydrogel, proper cell adhesiveness peptides, the degradation sequences, and the presentation of angiogenic signals is instrumental in promoting vascular morphogenesis in both controlled laboratory settings and living organisms. Cell–matrix interactions in vivo involve encountering different extracellular matrix proteins and their specific domains simultaneously. Studies have investigated the effects of combining multiple peptides on vascularization. For instance, in peptide-functionalized, degradable, PEG-based hydrogels, incorporating both YIGSR and RGDS results in the highest tubulogenesis and ECM protein production by encapsulated cells, which are hallmarks for proper angiogenesis. In an in vivo corneal experiment, the combination of RGDS, IKVAV, and YIGSR in a PEG hydrogel resulted in increased vessel density, branching, and other tubulogenic measures compared to using the peptides alone. These provide evidence for synergetic use of various cell–protein domain adhesion [169].

In contrast to earlier approaches that necessitated surgical implantation, a novel technique utilizes microgels created from PEG engineered to present RGD and VEGF. An MMP-degradable protein sequence was used to crosslink them. This innovation allows for the direct injection of microgels into mice, effectively triggering vascularization and facilitating tissue regeneration as the material gradually degrades over time [176].

In summary, attaching short peptides to hydrogel matrices provides better control over cell adhesion, allowing precise modulation of adhesive sequence presentation and density. Peptides such as RGD, IKVAV, and YIGSR have demonstrated their effectiveness in promoting cell adhesion, tubulogenesis, and vascularization, both in vitro and in vivo, offering promising opportunities for tissue engineering applications.

#### 3.2.2. Exploiting Hydrogel-Controlled Breakdown and Cell Migration for Improved Vascularization

Cell migration and the formation of new vascular networks are essential processes in tissue engineering. Macroporous hydrogels, such as cryogels, have been employed to support vascularization by providing large pores that allow cell spreading and migration, enabling vessel ingrowth [177,178,179,180]. However, in nanoporous hydrogels, cell migration through the gel network requires the degradation of the hydrogel material. Degradation allows cells to remodel their hydrogel environment and change the original materials with the ECM [181,182].

Efforts were made in the design of synthetic hydrogels, which can be remodeled as cells differentiate. Generally, synthetic gel biodegradation is obtained via hydrolysis and incorporation of proteolytic peptide sequences derived from ECM proteins [181,183]. Hydrolysis occurs when polymers linked by certain chemical groups undergo cleavage in aqueous environments. This process can be controlled by environmental factors and the hydrophilicity and permeability of the gel. Polymers prone to hydrolysis, like poly(lactic acid) and poly(glycolic acid), can be mixed with non-degradable polymers such as PEG to impart hydrolytic degradation to hydrogels [184,185]. However, hydrolysis is not directly responsive to cell behavior, limiting its control over tissue development.

To achieve cell-mediated and spatially controlled gel degradation, enzyme-degradable peptides derived from ECM proteins are often conjugated with synthetic polymers. Matrix metalloproteinases (MMPs) released by local cells during ECM remodeling are known to cleave in these sequences [186,187]. MMPs play a crucial role in tissue vascularization and are upregulated in various diseases. Different peptides have different susceptibilities to MMPs, and their inclusion in hydrogel designs allows for degradation rates controlled by cell behavior. Changing the number and sequence type also affects the degradation profile [181,188]. MMP-sensitive, collagen-derived peptide sequences were initially used for enzymatically degradable hydrogels, but modified versions have been developed to enhance degradation rates. Peptides can vary in their degradation rates and sensitivities to different MMPs, offering design flexibility for gels supporting vascularization. MMP-degradable peptide sequences, particularly those sensitive to MMP2 and MMP9 secreted by vascular cells, are commonly employed in vascularization support [189,190]. Studies have shown that optimizing the degradation rate of hydrogels influences vascular sprout formation and architecture. An intermediate degradation rate promotes multicellular migration, resulting in more complete sprout formation, while very fast or slow degradation rates hinder sprout connectivity and cell invasion [174,191].

In summary, the incorporation of degradable peptides into synthetic hydrogel matrices allows for cell-mediated gel degradation and controlled tissue remodeling. By responding to cell behavior and MMP activity, these hydrogels facilitate cell migration, ECM remodeling, and the formation of functional vascular networks.

#### 3.2.3. Exploiting the Angiogenic Factors-Controlled Release for Improved Vascularization

Angiogenesis is a tightly regulated and time-sensitive process that requires prolonged exposure to factors. While the soluble release of pro-angiogenic factors is important for recruiting vascular cells, delivering them in a single bolus is insufficient to sustain all the process due to rapid clearance and unintended side effects. Therefore, several strategies are used to allow either the retention or the controlled release of angiogenic growth factors. They include: the integration of protease degradable linkers, the heparin and aptamer binding to signaling molecules, and the entrapment in emulsion of angiogenic factors in micelles (Table 6).

- The use of degradable linkers: Besides aiding in the migration of encapsulated cells and promoting endogenous tissue growth, matrix degradation can be employed as a mechanism to regulate the release of angiogenic growth factors into the proper sites. [192,193]. When growth factors are released through the degradation of the gel, they are released over an extended period, which has been shown to enhance angiogenesis. In this scenario, studies have indicated that VEGFs were encapsulated within RGD-functionalized PEG microgels and crosslinked using either a degradable peptide, GCRDVPMSMRGGDRCG (VPM), that can be broken down by MMP-1 and MMP-2 enzymes, or a non-degradable linker, DTT. The speed of gel degradation was modified by varying the proportion between the enzymatically breakable VPM linkers and the enzymatically unaffected DTT crosslinkers. As expected, the regulated release of VEGFs resulted in a significantly increased number of blood vessels [176].
  Using a different strategy, the angiogenic peptides SPARC113 and SPARC118 were integrated into the gel structure, surrounded by MMP-cleavable regions. In vivo experiments demonstrated that gel degradation and the subsequent release of these peptides substantially boosted endogenous angiogenesis. These results suggest that by incorporating various cleavable regions, the matrix’s degradation rate can be controlled, allowing for the regulated release of VEGFs, which, in turn, is able to control vessel formation [194].
- The use of heparin binding: Another method for achieving prolonged release and presentation of angiogenic factors to cells is through heparin binding, which temporarily immobilizes biomolecules. This approach is due to the ability of heparin to bind GFs through electrostatic interactions [195,196]. This sequestration results in improved stability and gradual release of angiogenic factors such as VEGF and bFGF, which maintain their functions [142,197]. Heparin binding facilitates biomolecule presentation by mixing heparin with proteins in vitro. Covalently linked heparin-biomolecule complexes exhibit extended sustained growth factor release compared to non-covalent bonds in the polymer matrix. Studies with heparin-containing gels show reduced initial burst release and prolonged sustained release of pro-angiogenic factors in vitro for up to 21 days [198,199]. The extended duration of interaction has been discovered to amplify the angiogenic reaction of vascular cells in PVA-heparin gels, resulting in enhanced HUVEC migration when exposed to bFGF and VEGF separately, as well as with the simultaneous binding of both bFGFs and VEGFs [142]. Moreover, the in vivo implantation of hydrogel with heparin-bound GFs has demonstrated successful vascularization. Heparin-bound VEGFs have promoted the ingrowth of endogenous blood vessels either into degradable PE or gelatin-based hydrogels [88,200]. Similarly, poly(lactic-co-glycolic acid)-heparin microspheres loaded with bFGF have enhanced vascularization when implanted. Poly(lactic-co-glycolic acid)-heparin microspheres, when coupled with bFGFs, effectively increased the density of local capillaries in a subcutaneous model. Similarly, VEGF-bound hyaluronan-heparin gels stimulated angiogenesis in a subcutaneous context and supported the sustained formation of blood vessels for 28 days [197,198].
- The use of aptamers: Aptamers are short oligonucleotide strands exhibiting high specificity in binding proteins [82,197,198]. They can also be conjugated to hydrogel constituents. These molecules offer an advantage in biomaterial functionalization as they specifically bind to targets without inducing an immunogenic response [201,202]. The conjugation of aptamers, which are specific to pro-vascular factors, with polymer hydrogels has yielded angiogenic responses. As an example, the use of anti-VEGF aptamer binding VEGFs showed greater HUVEC growth in the presence of the anti-VEGF aptamer than the soluble VEGF [203]. Additionally, fibronectin gel possessing anti-VEGF and anti-PDGF aptamers exhibit a significant increase in ECs in vitro and boosted vessel numbers showing hallmarks of mature vascularization units in vivo [204]. Similarly, an aptamer-based programmable VEGF delivery platform was implemented in GelMA hydrogels and was used to tune the microvasculature formation within engineered tissues [202]. Thus, both heparin and aptamers can serve as effective means of binding multiple pro-angiogenic factors to enable prolonged exposure to cells, thereby enhancing the angiogenic response.
- The use of entrapment in emulsion of angiogenic factors: It represents an alternative strategy which enables the control of the release from gels of entrapped angiogenic factors, allowing for spatiotemporal regulation. Recently, a biomaterial was designed with ultrasound technology which enabled the synthesis of hydrogel-loaded, acoustically sensitive emulsions [205]. When subjected to ultrasound exposure, the emulsion underwent evaporation, leading to the release of bFGFs and inducing a controlled, time-dependent enhancement in endothelial cell tubule sprouting.

**Table 6:** Common methods for angiogenic-factor-controlled release.

Release Methods	Angiogenic Factors	References
Degradation	VEGF	[176]
Heparin binding	VEGF	[88]
	bFGF	[142,198]
	VEGF + bFGF	[142,198]
Aptamer binding	VEGF	[204]
	PDGF	[204]
	VEGF + PDGF	[204]
Emulsion entrapment	bFGF	[205]

## 4. Integration of the Angiogenic Niche Physiology within Smart Hydrogels

**Figure 3:**
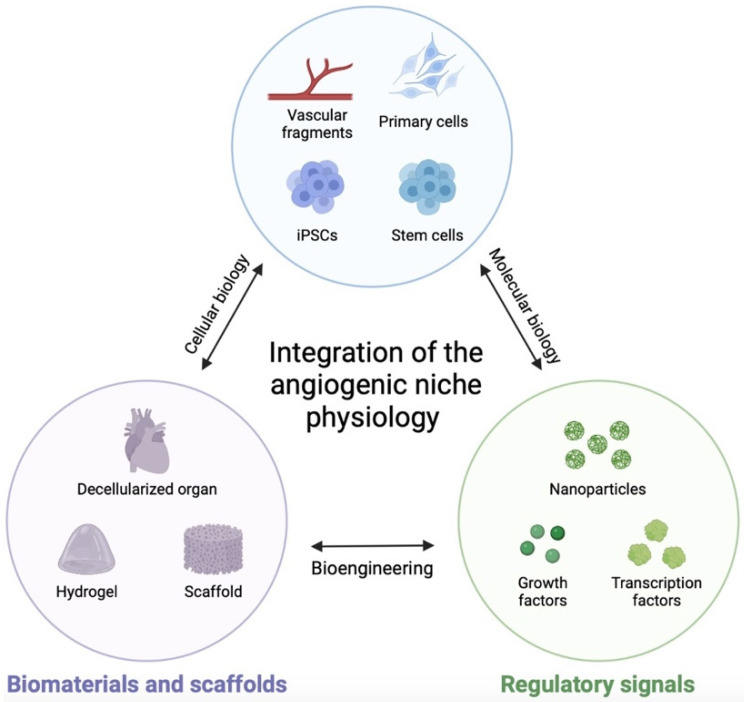
Integration of the angiogenic niche physiology is mandatory for the creation of smart hydrogels fully supporting tissue engineering and therapeutic regeneration. Combining expertise from different disciplines will provide suitable tools for tissue functionality and therapeutic outcomes.

Vasculature plays a crucial role not only as a conduit for nutrients and oxygen but also as a dynamic regulator of biological processes. In vitro studies that incorporate vascularized microenvironments offer researchers the opportunity to explore the interplay between diverse cell types; biophysical factors like shear stress; and extracellular matrix (ECM) organization in processes such as regeneration, stem cell maintenance and expansion, and disease progression. However, it is essential to validate biomaterial platforms using in vivo models to ensure that mechanistic findings have clinical relevance. Biomaterials designed to replicate an in vivo microenvironment should faithfully mimic specific aspects of the microenvironment, including mechanical properties, matrix composition, and cellular organization. In addition to engineering the ECM, there may be a need to engineer the vascular cells incorporated into the biomaterial. The transcriptomic and proteomic characteristics of vascular cells are influenced by their microenvironment, the health or disease state of the tissue, and the specific vascular sub-niche they inhabit (e.g., stable vasculature vs. active angiogenesis, tip vs. stalk) (Figure 3). Many biomaterial research studies employ commonly use vascular cell types such as HUVECs and MSCs, which may not inherently mimic the vascular cells found in a particular tissue, disease, injury state, or vascular environment/phenomena, even when provided with instructions from an engineered ECM [206]. Although it may be possible to isolate vascular cells from the desired tissue or pathophysiological model, their in vitro phenotype may still differ from their in vivo behavior.

Therefore, it may be necessary to program vascular cells to recreate essential transcriptomic or proteomic characteristics that mimic their in vivo counterparts. This can be achieved through genetic engineering and synthetic biology techniques, allowing the introduction, amplification, or knockdown of specific genes of interest [207]. Additionally, the ability to trigger transcriptomic or proteomic changes in vascular cells using light- or chemically induced constructs provides temporal control over cell behavior [208,209], which is particularly relevant for modeling transitions or the onset of pathophysiological states.

In summary, the successful development of an in vitro model can provide valuable insights for designing biomaterials with in vivo applications. In these applications, infiltrating vasculature not only provides nourishment to the tissue but also actively guides signaling for regeneration.

Understanding the impact of angiocrine signals on biological outcomes often requires the co-culture of different cell types, including vascular, parenchymal, and immune cells. When utilizing biomaterials in such studies, it becomes essential to incorporate methodologies that reveal signaling networks between these heterogeneous cell populations. One approach involves the use of single-cell RNA sequencing, which can identify potential receptor–ligand interactions at the transcriptome level [210]. Additionally, cell-specific proteomic labeling plays a vital role in detecting reciprocal changes in secreted and intracellular proteins, as well as deposited extracellular matrix (ECM) components [211,212,213]. To retrieve cells and proteomic samples without causing damage, strategies for biomaterial degradation post-culture need to be implemented [214]. The integration of next-generation sequencing and mass-spectrometry-based proteomics analysis often results in multidimensional datasets, where concurrent changes in multiple genes and proteins must be correlated with specific cell phenotypes in a meaningful way. Statistical analysis techniques, such as partial least squares regression, can be utilized to generate models that extract information from big data. Moreover, by employing systems biology strategies, such as computational modeling, network analysis, and omics data integration, researchers can gain valuable insights into the complex processes involved in vascularization, leading to improved strategies for constructing vascularized engineered tissues [215].

Collaborations between the fields of biomaterials, chemical biology, and bioinformatics are crucial for leveraging these tools effectively.

## 5. Future Directions

The wide range of cellular studies, tunable hydrogels, and preclinical applications reported in this review expand the possibility towards the development of 3D tissue models with improved vascularization. So far, extensive research has shown that smart hydrogels, as intended above, can mimic various organs, such as the bone, kidney, liver, lung, muscle, and brain. These accurate and reproducible 3D organoids have expanded the potential applications in several fields other than tissue regeneration, which include organ-on-chips and the development of personalized drug screening platforms. Preclinical studies have demonstrated the potential of hydrogel pre-vascularization as a promising method for tissue regeneration upon transplantation. However, conventional tissue transplantation possesses several drawbacks including complex surgical procedures, improper adaptation, and infection risks, which may cause the failure of the procedure. Injectable vascularized hydrogels offer a solution with improved defect margin adaptation, reaching deep tissues with minimal invasiveness and acting as carriers for GFs, cells, and drugs including antimicrobials. Thus, further studies will address the suitability of combining different bioactive molecules in a single pre-vascularized hydrogel. Another field of tremendous development is drug testing. Conventional drug assessment using 2D cell cultures and 3D animal models lacks accuracy and raises ethical and cost issues. Hydrogel-based tissue models are gaining interest for artificial organ development due to their customizable properties, permeability, and biocompatibility, and are also developing in the direction of tailored medicine.

## 6. Conclusions

The development of vascularized engineered tissues represents a remarkable breakthrough in the field of regenerative medicine. By creating functional tissues that possess an intricate network of blood vessels, researchers aim to address the limitations of traditional tissue engineering approaches. One promising avenue in this pursuit involves the design of smart hydrogels that can mimic signaling pathways, thereby promoting the formation of vascular networks within the engineered tissues. This review explores the significance of such an approach. Signaling pathways play a critical role in orchestrating complex cellular processes, including angiogenesis and the formation of new blood vessels. In native tissues, various signaling molecules, growth factors, and cytokines regulate the behavior of cells involved in angiogenesis. By deciphering the intricate interplay of these signaling pathways, researchers can emulate them within the design of smart hydrogels, thereby driving the formation of a functional vasculature.

Smart hydrogels are intelligent biomaterials that possess the ability to respond to specific stimuli. By incorporating signaling molecules and growth factors within the hydrogel matrix, researchers can create an environment that closely resembles the natural signaling cues required for vascularization (Figure 3). Additionally, the properties of the hydrogel, such as its mechanical and chemical characteristics, can be fine-tuned to promote the desired cellular behavior, including cell adhesion, migration, and differentiation. In the design of smart hydrogels for vascularized engineered tissues, researchers aim to replicate key signaling pathways involved in angiogenesis. One example is the vascular endothelial growth factor (VEGF) signaling pathway, which plays a central role in stimulating endothelial cell proliferation and migration. By incorporating VEGFs or VEGF-mimicking molecules within the hydrogel, researchers can promote the formation of new blood vessels within the engineered tissue construct. The use of smart hydrogels that mimic signaling pathways in the development of vascularized engineered tissues offers several therapeutic advantages. Firstly, the presence of functional blood vessels allows for improved nutrient and oxygen supply to the cells, enhancing their viability and function. This is particularly crucial for large tissue constructs, where diffusion alone is insufficient. Secondly, the vascular network facilitates the transport of immune cells, growth factors, and therapeutic agents, enabling efficient tissue repair and regeneration. Finally, the integration of smart hydrogels with signaling capabilities offers precise control over the formation and organization of blood vessels, allowing for the development of complex tissue architectures.

The use of naturally derived or synthetic hydrogel-based materials in fabricating perfusable models shows either advantages or disadvantages in terms of gelation, which greatly influences cell behavior. For example, it is recognized that collagen is pivotal in the design of hydrogel and fibrin hydrogels because it strongly supports vascularization; however, controlling collagen and fibrin gelation via temperature and pH often represents a challenge. Thus, gelatin and, more precisely, gelMA are usually addressed for successful vascularization due to their crosslinking possibility and chemical modifications. It is known that naturally derived polymers possess innate biocompatibility and show proper cell adhesion and cell-dependent degradation, allowing for hydrogel remodeling in a manner that resembles the ECM of a natural tissue. In addition, functional groups can be used for engineering via crosslinking, thus integrating other information to cells. However, batch-to-batch variability is the main disadvantage of hydrogels that are based on collagen, gelatin, fibrin, or HA. In addition, each component possesses typical features, as HA does not contain integrin-binding domains or MMP sites. Thus, modifications need to be taken into account so as to incorporate these sites. Moreover, it has been shown that HA interacts with cells through CD44 and RHAMM receptors, and its content is modified due to the action of HA synthases and hyaluronidases [216]. These disadvantages may be overcome because HA is highly receptive to chemical modifications, enabling the development of a hydrogel that supports cell adhesiveness and presents angiogenic factors [217]. Synthetic polymers are generally known for providing control in each step of preparation and possess high reproducibility. However, PVA- or PEG-based hydrogels usually require modifications for resembling an ECM environment and for proving functional cues to cells. Generally, as a schematic workflow pipeline, synthetic hydrogels can be utilized for mechanistic studies that investigate the impact of single matrix components and properties on angiogenesis. However, they do not fulfill the requirement for recapitulating the physiological niche because are not representative of the complexity of the ECM.

In this scenario, it should be noted that these two approaches are not mutually exclusive and can be integrated to generate smart hydrogels. As an example, researchers developed a scaffold containing PCL/collagen fibers electro-sprayed with HA and loaded with VEGFs and PDGF-BB [218], which combined all the advantages belonging to the different materials.

Therefore, it is reasonable that achieving a harmonious balance between material characteristics and adjustments, including the integration of GFs to any possible used procedure, becomes crucial when aiming for vascularization. In these terms, measuring the vascular length, density, volume, and the modification of ECM components [159,172] are common procedures for assessing the optimal strategies to be used.

The development of vascularized engineered tissues using smart hydrogels is a rapidly evolving field. Further research is required to refine the design and fabrication techniques, optimize the incorporation of signaling molecules within the hydrogel matrix, and enhance the long-term stability of the vascular network. Additionally, investigating the potential of other signaling pathways and their integration into smart hydrogels could open up new avenues for therapeutic applications. The development of vascularized engineered tissues holds immense promise in the field of regenerative medicine. By mimicking signaling pathways within the design of smart hydrogels, researchers can promote the formation of functional blood vessels and achieve therapeutic advantages. The integration of these vascular networks enhances nutrient supply, facilitates immune response and drug delivery, and allows for the development of complex tissue architectures. Continued advancements in this field will contribute to the realization of functional and clinically relevant tissue constructs, bringing us closer to a new era of regenerative medicine. In conclusion, ongoing advancements in engineering angiogenic biomaterials hold promise for enhancing the efficacy of regenerative therapies and providing deeper insights into the mechanisms underlying regeneration, stem cell behavior, and disease progression. These innovations will significantly impact the delivery and design of healthcare solutions for various injuries and contribute to the development of innovative treatments for a wide range of diseases, including neurodegeneration and cancer.

## Figures and Tables

**Table 5:** Common methods for improving cell adhesion.

Adhesive Peptides	Hydrogel	References
RGD	PEG	[172,173,174]
IKVAV	PEG	[175]
PEG-IKVAV	PEG	[175]
PEG-YIGSR	PEG	[175]
PEG-RGD	PEG	[175]
PEG-RGD + YIGSR + IKVAV	PEG	[169]

## Data Availability

Not applicable.
